# Interdisziplinäre Kommunikation: Augenarztbriefe an diabetologische Schwerpunktpraxen (DSP)

**DOI:** 10.1007/s00347-020-01179-2

**Published:** 2020-07-16

**Authors:** Lydia Stock, Daniel Roeck, Andreas Fritsche, Tjalf Ziemssen, Focke Ziemssen

**Affiliations:** 1 Center for Ophthalmology, Eberhard Karl University of Tuebingen, Tuebingen, Deutschland; 2 Division of Endocrinology, Diabetology, Vascular Disease, Nephrology and Clinical Chemistry, Department of Internal Medicine, University of Tuebingen, Tuebingen, Deutschland; 3 Center of Clinical Neuroscience, Department of Neurology, University Hospital Carl Gustav Carus, Dresden University of Technology, Dresden, Deutschland; 4 University Eye Hospital Tuebingen, Elfriede-Aulhorn-Str. 7, 72076 Tuebingen, Deutschland

**Keywords:** Arztbrief, Diabetische Retinopathie, Interdisziplinäre Kooperation, Elektronische Patientenakte, Digitalisierung, Physician’s letters, Diabetic retinopathy, Interdisciplinary communication, Electronic patient record, Digitalization

## Abstract

**Hintergrund:**

Arztbriefe und Befundbögen spielen für die interdisziplinäre Zusammenarbeit von Hausarzt, Internist, Diabetologe und Augenarzt als Informations- und Kommunikationsmittel in der Vermeidung, Verzögerung und Therapie der diabetischen Retinopathie (DR) eine zentrale Rolle.

**Methode:**

In einer Querschnittstudie (NCT02311504) wurden die augenärztlichen Briefe sowie weitere klinische Parameter aus der elektronischen Patientenakte (ePA) für 810 Patienten mit Diabetes extrahiert. Neben einer formalen Kategorisierung wurden die Dokumente auf ihre Aktualität und den inhaltlichen Aufbau hin überprüft und entsprechend den Themenkomplexen der nationalen Versorgungsleitlinie (NVL) für Netzhautkomplikationen analysiert.

**Ergebnisse:**

Für 59 % aller Patienten lag in den DSP ein augenärztlicher Befundbericht vor; 26 % der Dokumente wurden elektronisch generiert, 73 % handschriftlich verfasst; 55 % entsprachen dem Standardbogen der NVL, 21 % waren selbst entworfene Formularbögen der Augenarztpraxen, 16 % ausführliche Augenarztbriefe, 5 % Kurzbriefe und 3 % Kurzmitteilungen. Das durchschnittliche Alter des aktuellsten vorliegenden Berichts lag bei 19 Monaten. Ein Viertel der Dokumente war zum Zeitpunkt der Stichprobe älter als 2 Jahre; 75 % aller Patienten wurden in den letzten 12 Monaten augenärztlich betreut mit einer augenärztlichen Berichtsquote von nur 40 %. Die Prävalenz der berichteten DR lag für die Formulare bei 12 %, in ausführlichen Briefen bei 32 %.

**Schlussfolgerung:**

Obwohl standardisierte Formulare in der augenärztlichen Kommunikation mit den DSP einen hohen Verbreitungsgrad haben, kann die Berichtsquote verbessert werden, um eine zeitnahe Berücksichtigung relevanter Befunde zu ermöglichen. Die hohe Anzahl handgeschriebener Dokumente wies hier auf ein großes Potenzial elektronischer Formate in der interdisziplinären Kommunikation hin.

## Abstract (en)

**Background:**

Ophthalmologists’ letters and medical reports play an important role in the interdisciplinary cooperation between general practitioner, internist, diabetologist and ophthalmologist providing means of information and communication for the avoidance, delay and treatment of diabetic retinopathy (DR).

**Methods:**

In a cross-sectional study (NCT02311504) the ophthalmologicsts’ letters and other clinical parameters were extracted from electronic patient records (EPR) of 810 patients with diabetes. Files were classified into different categories of formats, tested with respect to topicality and content structure and analyzed according to the topic complex of the national treatment guidelines for retinal complications.

**Results:**

In 59% of the patients an ophthalmologist’s medical report was identified in the EPR. Of these, 26% were computer-generated documents, 73% were handwritten. 55% used the standard form of the national guidelines, 21% were self-designed formats, 16% detailed letters, 5% short reports and 3% short messages. The information was written on average 19 months ago. Of the documents 25% were older than 2 years at the time of the study. Of the patients 75% visited an ophthalmologist in the previous 12 months, yielding a report rate of only 40%. The prevalence of DR reported in the forms was 12%, in detailed letters 32%.

**Conclusion:**

Although standardized forms are widely distributed in the ophthalmologists’ communication with diabetologists, there is room for improvement in the face of the low report rate to enable a timely consideration of relevant findings. The high number of handwritten documents shows the large untapped potential of electronic formats in the interdisciplinary communication.

## Hintergrund

Über die Fachgrenzen hinweg ist der Arztbrief das klassische Instrument, das dem Informationsaustausch und der Kommunikation zwischen Ärzten dient [1, 2]. Für Menschen mit Diabetes mellitus (DM) kommt insbesondere auch der Übermittlung augenärztlicher Befunde an den Hausarzt, Internist oder Diabetologen eine besondere Bedeutung zu [3]. Ein Brief mit den relevanten Untersuchungsergebnissen kann Kosten für überflüssige Mehrfachdiagnostik einsparen und gerade die Qualität der Behandlung chronischer Erkrankungen verbessern [2].

Die 2015 revidierte Nationale Versorgungsleitlinie (NVL) sieht inzwischen einen Dialog in beide Richtungen vor, weil die Informationen der jeweils anderen Fachdisziplin große Relevanz für die Beurteilung der individuellen Risiken, aber auch das konkrete Vorgehen (Kontrollintervalle, Behandlungsempfehlungen) haben [4, 5].

In Aus‑, Fort- und Weiterbildung wird die schriftliche Kommunikation oft nur am Rand berücksichtigt [1]. Dabei sollten Sprache, formale Struktur und Inhalte auf die jeweilige Erkrankung und die Bedürfnisse des Briefempfängers optimiert werden [4, 5]. Im Rahmen dieser Arbeit sollte die versorgungswissenschaftliche Analyse erfassen, inwieweit Briefe und Dokumente des Augenarztes als Kommunikationsmittel mit Diabetologen genutzt werden.

## Methode

Die DiabCheck^OCTplus^-Studie war eine prospektive Querschnittstudie im Süden Deutschlands, in der Menschen mit nachgewiesenem Diabetes konsekutiv eingeschlossen wurden (NCT02311504). Um eine möglichst repräsentative Stichprobe zu erhalten, wurden aus 10 Arztpraxen 3 diabetologische Schwerpunktpraxen (DSP) ausgewählt, die in unterschiedlichen regionalen Wirtschaftsräumen lagen. Die erste DSP lag im Zentrum einer von Industrie und Dienstleistungen geprägten Großstadt (>500.000 Einwohner), eine weitere in einer großen Kreisstadt (50.000 EW) im Einzugsgebiet einer Metropolregion und die dritte im Mittelzentrum (25.000 EW) eines ländlich geprägten dünn besiedelten Raumes. Die Beobachtung der ambulanten Langzeitbetreuung, hier insbesondere die Steuerung der fachärztlichen Früherkennung (Screening) erfasste hier die hausärztliche Grundversorgung genauso wie die Sekundärversorgung der analysierten DSP [6–9]. Personelle Voraussetzung einer DSP ist ärztlicherseits ein/e Diabetologe/in sowie als Assistenz ein/e Diabetesberater/in [6].

Von 831 kontaktierten Patienten willigten 810 in die freiwillige Studienteilnahme ein und füllten einen für die Studie erstellten Fragebogen aus. Einschlusskriterien waren ein Alter über 18 Jahre, ein in der elektronischen Patientenakte (ePA) dokumentierter Diabetes mellitus sowie ein positives Einverständnis des Patienten für das Studium ihrer Krankenunterlagen. Ausschlusskriterien waren Demenz, geistige Behinderung oder mangelnde Deutschkenntnisse. Es lag ein positives Votum seitens der Ethik-Kommission der Universität Tübingen vor. Diabetesdauer und -typ, Laborwerte, Komorbiditäten sowie augenärztliche Dokumente wurden aus der ePA entnommen.

In einem Fragebogen wurden Angaben zu Häufigkeit des Augenarztbesuchs (<1, 1‑mal, 2‑mal, 3‑mal, 4‑mal, >4-mal pro Jahr), Regelmäßigkeit und Zeitpunkt der letzten Kontrolluntersuchung erfragt.

Als NVL-Formular wurde der verwendete IFDA/AGDA-Bogen bezeichnet [4, 6]. Es erfolgte eine Klassifizierung der Berichte in folgende Formatkategorien:

1. vorausgefüllte NVL-Standardformulare [5], die mit Briefkopf der DSP, Patientendaten, aktuellem HbA_1c_-Wert sowie Vermerk „bitte um Rücksendung“ den Patienten mitgegeben worden waren, und NVL-Standardformulare der Augenarztpraxis,
2. selbst entworfene Formularbögen der Augenarztpraxen
3. ausführliche Briefe, die Anamnese, sämtliche Augendiagnosen, Untersuchungsergebnisse, zusammenfassende Beurteilung und empfohlene Kontrollintervalle enthielten,
4. Kurzbriefe mit Beschränkung auf die Augendiagnosen, kurze Beurteilung und das Kontrollintervall
5. Kurzmitteilungen (ausschließlich zum Status der DR).

**Figure 1:**
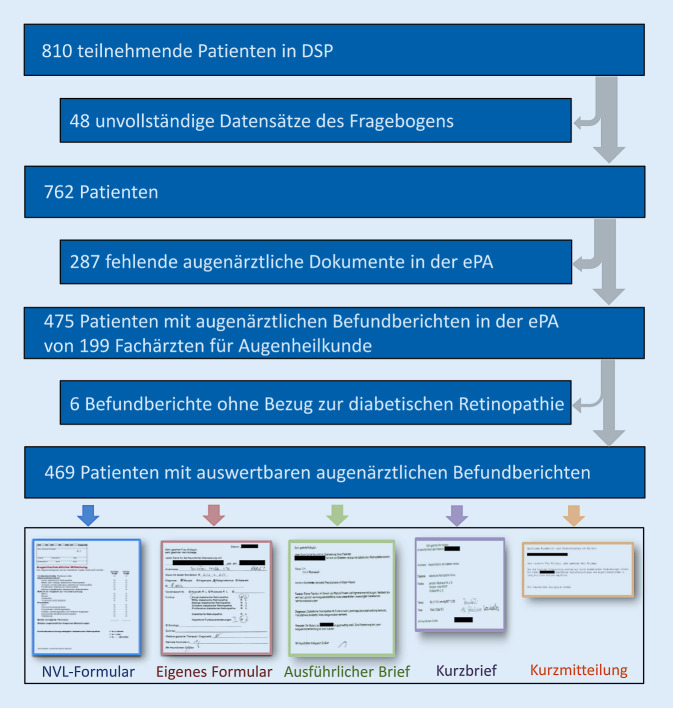

Analog zu den Inhalten der NVL von 2015 wurden folgende Themen analysiert [4]: Stadium der DR, bester Fernvisus, zusätzliche Diagnosen, Vergleich zu Voruntersuchungen, empfohlenes Vorgehen sowie Zeitintervall für Kontrolluntersuchung. Die Aktualität der Dokumente ergab sich aus dem Datum des letzten augenärztlichen Briefs an die DSP und dem Untersuchungszeitpunkt der Studie. Der Quotient aus der Anzahl der augenärztlichen Dokumente und der Anzahl der Augenarztbesuche pro Zeitintervall wurde als Berichtsquote nach einem Augenarztbesuch definiert. Unterschiede zwischen kategorialen Variablen wurden durch Anwendung des Chi-Quadrat-Testes ermittelt, stetige nicht normal verteilte Variablen mit dem Kruskal-Wallis-Test, normal verteilte mittels der Varianzanalyse. Assoziationen zwischen stetigen Variablen wurden mithilfe der multivariaten linearen Regressionsanalyse ermittelt. Ein *p*-Wert <0,05 wurde als statistisch signifikant bewertet. Die statistische Datenanalyse erfolgte mit SPSS 24 ([10]; Abb. 1).

## Ergebnisse

Das mittlere Alter der 810 Patienten betrug 58,7 (SD ± 15,4) Jahre (Tab. 1). Die berichteten Diagnosen (Tab. 2) stimmten nicht notwendigerweise mit den Anlässen der augenärztlichen Untersuchung überein. Mit einer Berichtsquote von 73,4 % wurden für Menschen mit Typ-1-Diabetes mellitus (T1DM) signifikant häufiger Briefe geschrieben als für Patienten mit Typ-2-Diabetes (T2DM, 50,9 %).

**Table 1:** 

Parameter		T1DM	T2DM
Prävalenz in der Studie *N* (%)		252 (31,1)	509 (62,8)
Anzahl der Briefe *n* (%)		185 (39,4)	259 (55,3)
Berichtsquote *n*/*N*		73,4	50,9
Mittleres Lebensalter/Jahre (SD)		48,0 (14,8)	66,1 (11,7)
Mittlere Diabetesdauer/Jahre (SD)		42,2 (13,8)	14,0 (8,3)
Prävalenz	DR	24,3 (45/185)	13,1 (34/259)
	Nephropathie	9,7 (18/185)	15,1 (39/259)
	Neuropathie	21,6 (40/185)	39,0 (191/259)
	Koronare Herzerkrankung	4,9 (9/185)	20,8 (54/259)

Stetige Variable: Mittelwert (SD)

**Table 2:** 

Diabetestyp (*n*)	T1DM (86)	T2DM (166)
Katarakt	24,4 (21/86)	42,8 (71/166)
Pseudophakie	15,1 (13/86)	18,1 (30/166)
Glaukom	3,4 (3/86)	7,2 (12/166)
Altersbedingte Makuladegeneration	3,4 (3/86)	11,4 (19/166)

Für 469 von 810 Patienten konnten die augenärztlichen Dokumente ausgewertet werden. Die Befunde wurden von insgesamt 199 verschiedenen Augenärzten aus 145 Augenarztpraxen übermittelt. Sämtliche augenärztliche Dokumente lagen als eingescanntes Dokument des Originals in der ePA der DSP vor.

### Form und Struktur

**Figure 2:**
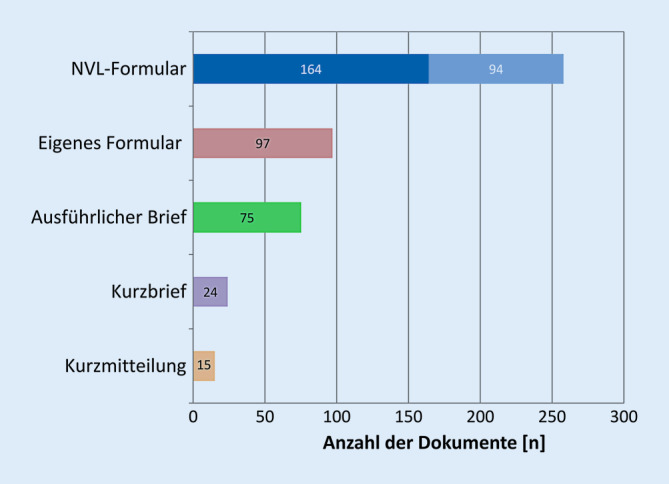

26,0 % (122/469) der Dokumente wurden elektronisch generiert, davon zwei Drittel als ausführliche Briefe und 1 von 5 mit vorbestehenden Formularen; 74,0 % (347/469) der Berichte wurden handschriftlich verfasst. Während praxisintern Form und Struktur in der Regel einheitlich gestaltet waren, zeigten sich zwischen den einzelnen Augenärzten erhebliche formelle Unterschiede (Abb. 2). Das breite Spektrum der augenärztlichen Mitteilungen erstreckte sich von Kurzmitteilungen auf einfachen roten Rezeptformularen bis hin zu ausführlichen mehrseitigen Augenarztbriefen; 55,0 % (164 + 94) aller Dokumente entsprachen dem Format des NVL-Formulars; 35,0 % (164) davon wiederum stellten durch die DSP vorausgefüllte NVL-Formularbögen, 20,0 % (94) NVL-Formularbögen aus den Augenarztpraxen dar; 20,7 % (97) waren selbst entworfene Formulare der Augenarztpraxen; 16,0 % (75) lagen in Form ausführlicher Arztbriefe, 5,1 % (24) als Kurzbriefe und 3,2 % (15) als Kurzmitteilungen vor.

Es wurden 75,7 % (355) aller Dokumente mithilfe strukturierter Formulare erstellt [4, 5].

### Aufbau und inhaltliche Gewichtung

In Bezug auf den inhaltlichen Aufbau ergaben sich signifikante unterschiedliche Gewichtungen in den Themenkomplexen (Tab. 3).

**Table 3:** 

		Stellungnahme zu den Themenkomplexen						
Struktur und Form der Dokumente (In der Tabelle explizit aufgeführt sind Dokumentenformen mit *n* > 15)	Anzahl *n*	DR-Stadium	Bester Fernvisus	Weitere augenärztliche Diagnosen neben der DR	Befundvergleich zur Voruntersuchung	Procedere		Angabe des Kontrollintervalls
						Stabiler Befund, weitere Diagnostik oder Therapie ist nicht erforderlich	Diagnostik- und Therapievorschlag	
		Quote in % (Anzahl *n*)						
*Standardformularbogen, von der DSP vorausgefüllt*	164	97,6 (160)	92,7 (152)	43,3 (71)	62,8 (103)	0,6 (1)	3,0 (5)	88,4 (145)
*NVL-Standardformular Augenarzt*	94	97,9 (92)	95,7 (90)	52,1 (49)	57,4 (54)	2,1 (2)	1,1 (1)	95,7 (90)
*Eigenformular*	97	100 (97)	55,7 (54)	67 (65)	26,8 (26)	19,6 (19)	2,1 (2)	91,8 (89)
*Ausführliche Briefe*	75	100 (75)	98,7 (74)	80,0 (62)	33,3 (25)	41,3 (31)	10,7 (8)	77,3 (58)
*Kurzbriefe*	24	100 (24)	75 (18)	79,2 (19)	29,2 (7)	12,5 (3)	12,5 (3)	33,3 (8)
*Kurzmitteilungen*	15	100 (15)	0	20 (3)	6,7 (1)	6,7 (1)	0	13,3 (2)
*Stellungnahme in allen augenärztlichen Dokumenten [%]*	*N* *=* *469* Gesamtzahl	*∑* *=* *98,7* (463/469)	*∑* *=* *82,7 *(388/469)	*∑* *=* *57,4* (269/469)	*∑* *=* *46,1* (216/469)	*∑* *=* *12,2 *(57/469)	*∑* *=* *4,1 *(19/469)	*∑* *=* *83,6* (392/469)
*Signifikanzniveau p*	–	<0,001	<0,001	<0,001	<0,001	<0,001		<0,001

In 98,7 % der Dokumente (463/469) wurden Angaben zum Retinopathiestadium gemacht. In 82,7 % (388/469) waren Angaben zum bestkorrigierten Fernvisus enthalten. In 57,4 % (269/469) gab es einen Abschnitt zu weiteren ophthalmologischen Diagnosen, wobei diese Information mit 80,0 % (62/75) am häufigsten in ausführlichen Briefen enthalten war. Für 46,1 % (216/469) wurden Befundvergleiche (Zunahme, Stabilität, Verbesserung) zur Voruntersuchung beschrieben. In 16,2 % der Dokumente (76/467) wurden Angaben zur weiteren Therapie und Diagnostik gemacht. Ein Vermerk „stabiler Befund, weitere Diagnostik oder Therapie nicht erforderlich“ wurde in 12,2 % der Arztbriefe (57/469) aufgenommen, besonders häufig in ausführlichen Briefen oder den selbst entworfenen Formularbögen. Eine Befundverbesserung wurde in 3 Fällen angegeben.

Eine klinische Konsequenz aus der augenärztlichen Untersuchung hinsichtlich Therapie und Diagnostik konnte aus mindestens 4 % (19/469) abgelesen werden. Die klinische Therapieempfehlung war bei 1,9 % (9/469) die Empfehlung einer Kataraktoperation oder Kapsulotomie, bei 1,5 % (7/469) eine Laserkoagulation oder IVOM. Als Diagnostik wurde für 0,6 % (3/467) eine Fluoreszenzangiographie bzw. OCT-Untersuchung angeraten. In 3 Fällen war ein diabetisches Makulaödem (DMÖ) zu behandeln, in einem Fall erfolgte die Überweisung in eine Universitäts-Augenklinik.

In 83,6 % (392/469) wurden präzise Zeitintervalle angeratener Kontrolluntersuchungen angegeben; 80,9 % der empfohlenen Kontrollintervalle (317/392) entsprachen in ihrem Abstand den empfohlenen Screeningintervallen der seinerzeit aktuellen NVL: Bei 7,4 % (29/392) wurden trotz eines unauffälligen Augenbefunds, normaler Visusangaben (VF ≥0,8) sowie fehlender begleitender Risikofaktoren halbjährliche Kontrollen vorgeschlagen.

### Aktualität

**Figure 3:**
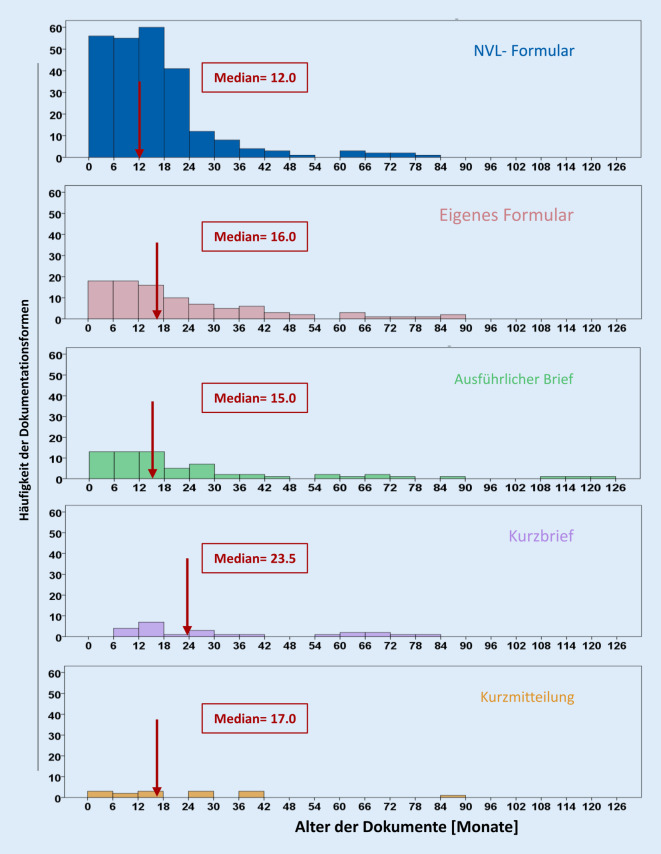

Die vorliegenden Dokumente waren zum Zeitpunkt der aktuellen diabetologischen Untersuchung im Mittel 19,1 Monate (95 %-KI: 17,3; 20,8) alt. Der Median für NVL-Formulare betrug 12,0, für selbst entworfene Formulare 16,0, für ausführliche Briefe 15,0, für Kurzbriefe 23,5, für Kurzmitteilungen 17,0 (Abb. 3). Ein Viertel aller Dokumente waren zum Zeitpunkt der Stichprobe bereits älter als 2 Jahre.

### Berichtsquote nach Augenarztbesuch

Es hatten 74,9 % (571/762) der Patienten in den 12 Monaten zuvor einen Augenarzt aufgesucht. Für 91,5 % (697/762) war eine Untersuchung in den letzten 24 Monaten dokumentiert.

Allerdings wurde nur für 39,6 % (226/571) nach dem augenärztlichen Besuch innerhalb der letzten 12 Monate ein Dokument angetroffen, bei 51,1 % (356/697) in den letzten 24 Monaten.

Wenn die NVL-Standardformulare bereits durch die DSP vorausgefüllt waren und den Patienten für den Augenarzt mitgegeben wurden, war die Berichtsquote mit 51,7 % (134/259) höher im Vergleich zum alternativen Vorgehen mit 29,5 % (92/312).

### Wahl der Dokumente bei DR

Die Prävalenz der DR lag in den Augenarztberichten für T1DM bei 24,3 % (45/185), für T2DM bei 13,2 % (34/258). Eine DR wurde in 11,6 % (30/258) der Standardformulare, in 19,6 % (19/97) der selbst entworfenen Formulare und in 32,0 % (24/75) der ausführlichen Briefe dokumentiert.

**Figure 4:**
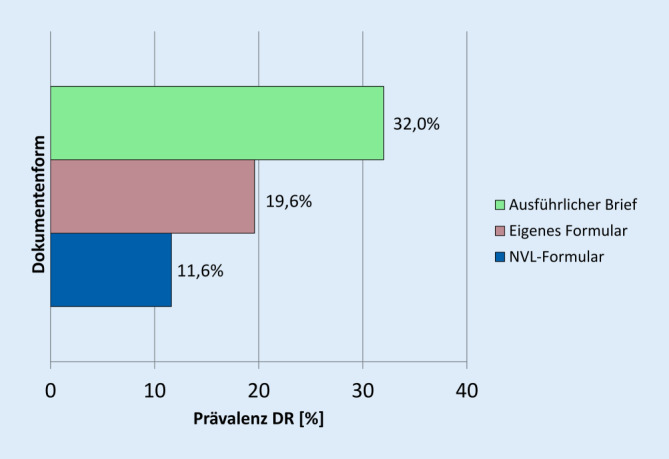

Der Anteil von ausführlich ausformulierten Briefen betrug 12,8 % (49/384) bei fehlender DR, 26,2 % (16/61) bei milder oder moderater DR und 45,4 % (10/22) bei fortgeschrittener DR oder DMÖ (Abb. 4).

## Diskussion

In der untersuchten Stichprobe wurde für die Patienten eine heterogene Berichtsqualität gefunden. Ophthalmologen muss bewusst sein, dass Hausärzten und Diabetologen nicht immer ein aktueller Befundbericht vorliegt. Für die Interpretation muss zum einen berücksichtigt werden, dass Patientenprofil und leitlinienkonforme Therapie auf eine Selektion hinweisen können, d. h. also eine überdurchschnittlich gute Betreuung durch die in der Studie beobachteten Diabetologen [7]. Zum anderen liefert die Erfassung über die ePA aufseiten der Hausärzte und Diabetologen keine Informationen darüber, wie viele Augenuntersuchungen tatsächlich erfolgten und nur aufgrund eines Übermittlungsfehlers der Dokumente nicht bekannt waren.

Während ein Teil der beobachteten Assoziationen nachvollziehbar war – je fortgeschrittener der Befund, desto zuverlässiger die Rückmeldung –, gab es konkrete Hinweise auf wahrscheinliche Hürden.

In einer repräsentativen DEGS1-Studie des Robert Koch-Instituts hatten 66,8 % der Patienten in den letzten 12 Monaten einen augenärztlichen Kontakt [8], in unserer Studie waren es 74,9 %. Diese Angabe dürfte eher noch geringer sein, da in die Auswertung auch Glaukompatienten mit einbezogen waren. Obwohl die augenärztliche Versorgung sehr stark von der Altersstruktur der Population [9] abhängt, waren die Ergebnisse in ihrer Größenordnung vergleichbar und dürfen als Teilerfolg der etablierten Disease-Management-Programme zum Screening diabetischer Augenveränderungen gesehen werden [11–13]. Allerdings weisen die Ergebnisse auch auf eine mögliche Unzuverlässigkeit und Unvollständigkeit einzelner Berichte der Augenkomplikationen hin. Somit dürfte es nicht ausreichen, sich in Studien allein auf die Erfassung von Ereignissen („incident reporting: event of special interest oder adverse event“) zu verlassen [14]. Eine systematische Dokumentation objektiver Befunde z. B. mittels Fotografien und die Absicherung durch zertifizierte Reading-Center wird immer überlegen sein [15].

Die interdisziplinäre Zusammenarbeit von Hausarzt, Internist, Diabetologe und Augenarzt kann weiter verbessert werden: Obwohl drei Viertel aller Patienten in den letzten 12 Monaten augenärztlich betreut wurden, lag die daraus resultierende augenärztliche Berichtsquote (Zeitraum von 12 Monaten) in der vorliegenden Studie nur bei 40 %. Vorausgefüllte Standardformulare, die den Patienten direkt mitgegeben wurden, erzielten eine deutlich höhere Berichtsquote der Augenärzte. Mitarbeit und Interesse der DSP an Befunden erhöhte möglicherweise die augenärztliche Bereitschaft, Briefe zu erstellen.

Sämtliche Ergebnisse dieser Studie beruhten auf augenärztlichen Dokumenten, die in der ePA vorgefunden wurden. Bei der Befundübermittlung sollte berücksichtigt werden, dass Befunde z. B. therapiebedürftiger Makulaödeme selten auch telefonisch übermittelt werden könnten oder eine mündliche Weitergabe an Patienten genutzt wurde. Möglicherweise wurde nicht jede augenärztliche Mitteilung als Original in die ePA eingescannt und nur das Untersuchungsergebnis in der ePA vermerkt.

Defizite in der augenärztlichen Kommunikation mit DSP zeigten sich auch in der geringen Aktualität der augenärztlichen Dokumente mit einem durchschnittlichen Alter von 19 Monaten. Ein Viertel aller vorgefundenen Dokumente war zum Zeitpunkt der Stichprobe sogar älter als 2 Jahre.

Der 1990 eingeführte Screening-Bogen der IFDA/AGDA (Initiativgruppe zur Früherkennung diabetischer Augenerkrankungen/Arbeitsgemeinschaft Diabetes und Auge [26]) wurde immer wieder überarbeitet und ist mittlerweile als augenfachärztlicher Untersuchungsbogen Bestandteil der NVL geworden [4, 5, 16]. Obwohl diese NVL-Standardformulare einen hohen Verbreitungsgrad von mehreren 100.000 Exemplaren erzielten [13], war die augenärztliche Kommunikation mit den DSP nicht einheitlich. Form und Struktur unterschieden sich deutlich. Die Standardformulare aus der NVL hatten mit einem Anteil von 55,0 % den höchsten Verbreitungsgrad. Auch die unterschiedlichen Formate machen es nicht einfacher, mit einer Botschaft fachfremde Kollegen zu erreichen.

Der Schriftverkehr mit den DSP wurde in 73,8 % der Fälle analog handschriftlich erstellt; 26 % der augenärztlichen Dokumente wurden digital z. B. mit Textverarbeitungsprogrammen generiert. Es ist zu erwarten, dass dieser Anteil in den letzten 5 Jahren noch angestiegen ist. Dennoch nutzte ein Großteil der Augenärzte den Vorteil der raschen Erstellbarkeit ohne Korrekturlesen bei standardisierten analogen Formularen.

Eine Analyse der im Auftrag des Digitalverbands Bitcom und des Hartmannbundes 2017 durchgeführten Befragung von 477 Ärzten ergab, dass 47 % der teilnehmenden Ärzte den innerärztlichen Schriftverkehr überwiegend handschriftlich gestalteten [17]. Mit der Entwicklung des elektronischen Arztbriefes (eArztbrief) beginnt derzeit die digitale innerärztliche Kommunikation [18, 19]. Dennoch gibt es relevante Hürden und organisatorischen Aufwand in der Gestaltung der Schnittstellen, wie die ungelösten Probleme und unerfreuliche Entwicklung der Telematikinfrastruktur belegen. Bisher verursachen papiergebundene Formate aber auch einen erheblichen Zeitaufwand, z. B. in Form des Scannens und des Imports in die ePA. Medienbrüche zwischen analoger und digitaler Welt forderten bei der Archivierung eine arbeitsintensive Nachbearbeitungszeit [3]. Außerdem ist das Vorliegen einer Information nicht mit der Kenntnisnahme gleichzusetzen, wenn Ärzte sich durch Historien unterschiedlicher Dokumente klicken müssen. Die hohe Anzahl handgeschriebener Dokumente war ein Hinweis auf das Potenzial der künftigen interdisziplinären Kommunikation und Effizienzreserven. Die Digitalisierung der augenärztlichen Kommunikation sollte kein Selbstzweck sein, sondern muss gegenüber der analogen Form einen spürbaren Nutzen bieten. Digitalisierte Augenarztbriefe müssen sich durch Zeitersparnis bei zumindest gleichem Standard in Rechtssicherheit und Datenschutz auszeichnen [20].

Die Standardisierung der Formulare kann angesichts der möglichen Vielfalt einer DR auch Grenzen haben. Vermutlich deshalb wurde mit Zunahme des Retinopathiegrades ein höherer Anteil von ausführlichen Briefen gefunden. Normalbefunde hingegen wurden bevorzugt (82 %) mit Formularen dargestellt. Die Analyse der inhaltlichen Gewichtung ergab bezüglich des Retinopathiestadiums, Visus, weiterer Diagnosen und Kontrollintervalle eine hohe Umsetzungsrate von über 80 %. Ein Vergleich zum Vorbefund wurde mit 46 % nur selten umgesetzt. Das war möglicherweise darin begründet, dass es sich beim IFDA/AGDA-Formular um einen Screeningbogen handelt, der sich v. a. für Normalbefunde oder milde Formen der DR eignet. Für fortgeschrittene Formen der DR oder gar zur Charakterisierung des DMÖ müssen die Limitationen des Bogens berücksichtigt werden. Es wurden 96 % der Befunde als unverändert zum Vorbefund beschrieben. Es könnte daher in den Standardformularbrief der NVL die Formulierung: „stabiler Befund, weitere Diagnostik oder Therapie ist nicht erforderlich“ mit aufgenommen werden.

Es zeigte sich nur eine geringe klinische Konsequenz aus den vorliegenden Berichten, denn therapeutische Eingriffe waren zur Behandlung der DR nur bei 1,5 % (Zeitfenster 4 Jahre) aller Patienten enthalten. Diese Größenordnung ist vergleichbar mit Studien aus Wales mit einer Therapierate von 1,2 % innerhalb von 4 Jahren [21] und England mit 1,3 % innerhalb von 5 Jahren [22]. Die geringe Komplikationsrate kann für die Bedeutung und den Erfolg einer intensiven Mitbehandlung von Risikofaktoren für eine DR, wie beispielsweise Hyperglykämie und arterielle Hypertonie, in den DSP sprechen. Zum anderen waren die zahlreichen ophthalmologischen Begleitdiagnosen ein Hinweis auf gründliche augenärztliche Untersuchungen, die über ein reines DR-Screening weit hinausgingen oder auch auf andere Probleme der Patienten zurückgingen [9].

Als Limitationen der Studie muss auf die Durchführung 2014 aufmerksam gemacht werden. Wie bereits erwähnt, wurde die NVL zwischenzeitlich revidiert. Die Verbreitung elektronischer Kommunikationswege dürfte inzwischen zugenommen haben, die zeitliche Belastung durch patientenferne Tätigkeiten und Bürokratie in der Arztpraxis leider auch [23]. Verglichen mit dem Primärbereich der hausärztlichen Versorgung wurden in den DSP (Sekundärebene) v. a. Patienten betreut, die einer intensiven Betreuung bedurften (T1DM; komplexe Therapie bei T2DM) [7]. Eine Übertragbarkeit auf alle Patienten mit DM ist daher nur eingeschränkt möglich. Der geringe Umfang der Stichprobe macht eine generelle Übertragung auf Deutschland nur bedingt möglich.

In Zukunft könnten intelligente Algorithmen die Erstellung und Analyse elektronischer Arztbriefe erleichtern, ohne dass generell auch die Verständlichkeit für die betroffenen Patienten leiden muss [24, 25]. Text dürfte nur noch in der Diagnoseübersicht sowie der zusammenfassenden Beurteilung zu finden sein. Elektronische Arztbriefe könnten nach Text und Bild aufgesplittet separat in einer auch für den Patienten und anderen behandelnden Ärzten zugänglichen, mehrfach gesicherten Cloud abgespeichert werden, um jederzeit verfügbar und abrufbar zu sein. Eine rasche und flächendeckende Ablösung analoger Formulare durch digitalisierte Augenarztbriefe oder Formulare verspricht dann einen klaren Nutzen, wenn Zeitersparnis bei zumindest gleichwertiger Rechtssicherheit und ausreichendem Datenschutz erreicht wird.

## Fazit für die Praxis

- Dokumente in Formularform zeigten einen hohen Verbreitungsgrad.
- Ausführliche Briefe wurden v. a. bei Patienten mit DR eingesetzt, Standardformulare bei Normalbefunden.
- Defizite in der augenärztlichen Kommunikation mit DSP zeigten sich in einer niedrigen Berichtsquote und geringen Aktualität der augenärztlichen Dokumente.
- Wenn Standardformulare durch die DSP vorausgefüllt wurden, sie an die Patienten mitgegeben wurden, erhöhte sich die Berichtsquote der Augenärzte deutlich.
- Für die Mehrzahl der Augenärzte waren die regelmäßigen Kontrolluntersuchungen bei DM nicht auf ein reines DR-Screening beschränkt.
- Die hohe Anzahl handgeschriebener Dokumente war ein Hinweis für ein großes Digitalisierungspotenzial in der augenärztlichen interdisziplinären Kommunikation.

## References

[1] Unnewehr M, Schaaf B, Friederichs H (2013). Arztbrief: Die Kommunikation optimieren. Dtsch Arztebl.

[2] Spießl H, Cording C (2001). Kurz, strukturiert und rasch übermittelt – Der „optimale“ Arztbrief. Dtsch Med Wochenschr.

[3] Hänel P, Herrmann M (2016). Kommunikation an Schnittstellen. Allgemeinchirurgische Patienten in der Hausarztpraxis.

[4] https://www.leitlinien.de/mdb/downloads/nvl/diabetes-mellitus/dm-netzhautkomplikationen-2aufl-vers2-lang.pdf. Zugegriffen: 28. Dez. 2019

[5] Bertram B (1999). Zusammenarbeit von Hausarzt und Augenarzt in der Diabetikerbetreuung Kommunikation unerläßlich. Dtsch Arztebl.

[6] Nauck MA, Meier JJ (2013). Kursbuch Klinische Diabetologie:Kurs-und Prüfungsinhalte der Weiterbildung zum Diabetologen (DDG).

[7] Marahrens L, Röck D, Ziemssen T, Kern R, Ziemssen F, Fritsche A (2017). Umsetzung der Nationalen VersorgungsLeitlinie (NVL) zur Therapie des Diabetes mellitus Typ 2 in diabetologischen Schwerpunktpraxen. Diabetes Aktuell.

[8] Kreft D, McGuinness MB, Doblhammer G, Finger RP (2018). Diabetic retinopathy screening in incident diabetes mellitus type 2 in Germany between 2004 and 2013—A prospective cohort study based on health claims data. PLoS ONE.

[9] Schuster AK, Wolfram C, Bertram B (2018). Wer geht wie oft zum Augenarzt in Deutschland? Ergebnisse der Studie zur Gesundheit Erwachsener in Deutschland (DEGS1). Ophthalmologe.

[10] IBM Corp. (2015). IBM SPSS Statistics for Windows, Version 24.0.

[11] Bertram B, Gante C, Hilgers RD (2013). Zunahme der Untersuchungen wegen Katarakt, Glaukom, diabetischer Retinopathie und Makuladegeneration: Vergleichende Querschnittstudie der Jahre 2010 und 1997 in Augenarztpraxen. Ophthalmologe.

[12] Rattay P, Butschalowsky H, Rommel A (2013). Inanspruchnahme der ambulanten und stationären medizinischen Versorgung in Deutschland. Bundesgesundheitsblatt Gesundheitsforschung Gesundheitsschutz.

[13] Initiative für diabetesbedingte Augenerkrankungen (IFDA) Initiative für diabetesbedingte Augenerkrankungen (IFDA). https://www.diabetes-auge.de/images/downloads/IFDA_Verein_2015.pdf. Zugegriffen: 20. April 2020

[14] Hoffmann B, Rohe J (2010). Patient safety and error management—What causes adverse events and how can they be prevented?. Dtsch Arztebl Int.

[15] Schmitz-Valckenberg S, Kühlewein L, Waldstein SM, Spital G, Ziemssen F, Liakopoulos S (2020). Zweitbeurteilung der retinalen Bildgebung. Ophthalmologe.

[16] https://www.diabetes-auge.de/index.php/informationen-fuer-aerzte/augenbogen. Zugegriffen: 10. Apr. 2020

[17] https://www.bitkom.org/Presse/Anhaenge-an-PIs/2017/06-Juni/Praesentation-170608-Aerztestudie-Koop-Hartmannbund-final.pdf. Zugegriffen: 10. April 2020

[18] Hillienhof A. Ärzteblatt (2017) Vorteile des E‑Arztbriefes bewähren sich in der Versorgung. https://www.aerzteblatt.de/nachrichten/83518/Vorteile-des-E-Arztbriefes-bewaehren-sich-in-der-Versorgung. Zugegriffen: 10. Apr. 2020

[19] Krüger-Brand HE (2012) Elektronischer Arztbrief – Im Praxisalltag angekommen. Dtsch Arztebl 109(21): A-1068 / B-920 / C-912

[20] Möller KH, Makosi K Der Arztbrief – Rechtliche Rahmenbedingungen. https://www.krvdigital.de/KrV.05.2015.186. Zugegriffen: 28. Dez. 2019

[21] Thomas RL, Dunstan F, Luzio SD (2012). Incidence of diabetic retinopathy in people with type 2 diabetes mellitus attending the Diabetic Retinopathy Screening Service for Wales: retrospective analysis. BMJ.

[22] Jones CD, Greenwood RH, Misra A, Bachmann MO (2012). Incidence and progression of diabetic retinopathy during 17 years of a population-based screening program in England. Diabetes Care.

[23] Korzilius H (2016). Bürokratie in der Arztpraxis: 52 Millionen Stunden für Papierkram. Dtsch Arztebl.

[24] Vitt KD, Erben CM, Dreimann M, Rüther W (2005). Patientenbrief: Mittel zur Sicherung des Heilerfolgs. Dtsch Arztebl.

[25] Vitt KD, Erben CM, Kupsch S, Rüther W (2008). Patientenbrief: Nachhaltige Information für Patienten. Dtsch Arztebl.

[26] Kroll P, Bertram B (1997). Augenfachärztlicher Untersuchungsbogen zur Früherkennung diabetischer Augenerkrankungen. Z Prakt Augenheilkd.

